# MitraClip as a therapeutic strategy for post-myocardial infarction mitral regurgitation

**DOI:** 10.1186/s13019-023-02165-w

**Published:** 2023-02-02

**Authors:** Anwu Huang, Ying Chen, Yiwei Huang, Xiaojun Ji, Wenbing Jiang

**Affiliations:** Department of Cardiology, Wenzhou Central Hospital, The Second Affiliated Hospital of Shanghai University, No. 252 Baili East Road, Wenzhou, 325000 Zhejiang Province People’s Republic of China

**Keywords:** Acute myocardial infarction, Mitral regurgitation, MitraClip, Edge-to-edge repair, Case report

## Abstract

Mitral regurgitation is a serious complication of post-myocardial infarction, with increasing mortality. Surgery as the primary treatment carries a high risk. MitraClip is a new therapeutic to treat post-myocardial infarction mitral regurgitation. In this case, a 62-year-old male patient suffered from severe heart failure symptoms after emergency coronary intervention and extracorporeal membrane oxygenation support. Based on cardiac echocardiography, severe mitral regurgitation was monitored in this patient. After MitraClip treatment, the patient's condition was gradually improved and discharged successfully. This case highlights that MitraClip is a safe and effective strategy for post-myocardial infarction mitral regurgitation.

## Introduction

Acute mitral regurgitation, a serious mechanical complication, is generally occurred within two to seven days after acute myocardial infarction [1]. It results from papillary muscle and chordae tendineae rupture or displacement of the apical and inferior of the papillary muscle due to remodeling of the infarcted left ventricle. Generally, surgical mitral valve repair or replacement is the primary treatment for mitral regurgitation [2]. However, these patients are often rejected surgery because of some possible serious events such as death [3]. Percutaneous edge-to-edge repair via MitraClip is recently a new therapeutic to treat post-myocardial infarction mitral regurgitation. Applying this technique can effectively ameliorate heart failure symptoms and reduce mortality. In this case, we report a patient with severe heart failure symptoms after emergency coronary intervention and extracorporeal membrane oxygenation (ECMO) support and that was managed successfully with edge-to-edge repair with the MitraClip system.

## Case presentation

**Fig. 1:**
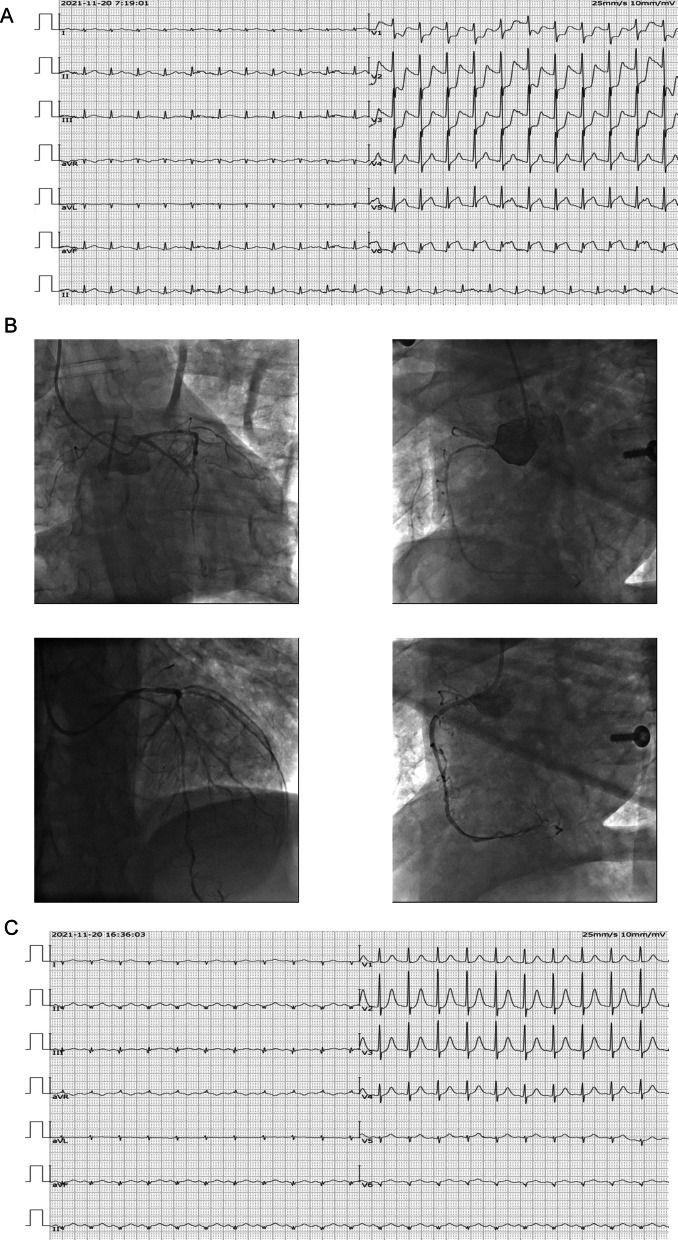
**A** Preoperative electrocardiogram (ECG) suggested acute myocardial infarction. **B** Coronary angiography suggested three coronary artery lesions, and A stent was respectively implanted in the left circumflex artery (LCX) and right coronary artery (RCA). **C** Postoperative ECG

A 62-year-old male patient with prior history of hypertension, diabetes, and diabetic nephropathy with symptoms of a sudden chest tightness that could not be relieved, along with nausea, vomiting and profuse sweating. He was urgently admitted to a nearby hospital for treatment. The blood pressure was 81/53 mmHg, and the oxygen saturation was only 67%. During the period, the patient suffered a sudden unconsciousness and then cardiac arrest. After immediate cardiopulmonary resuscitation, tracheal intubation and ventilator assisted ventilation treatments, the heart rate was recovered to 50–60 beats/min. Electrocardiogram (ECG) examination indicated the inferior ST-segment elevation and anterior ST-segment elevation, and the patient was immediately transferred to the emergency department of our hospital because these critical conditions. In our hospital, ECG re-examination showed sinus rhythm, ST depression in V1–V4 leads, and ST elevation in inferior and V5–V6 leads (Fig. 1A), which was diagnosed as acute myocardial infarction. Emergency coronary angiography (CAG) was performed after ventilator assisted ventilation, norepinephrine boosts pressure and sodium bicarbonate acid correcting. Baseline CAG showed patients with multivessel disease, manifesting as 70% diameter stenosis in the left anterior descending artery, totally occluded in the proximal left circumflex artery (LCX), and 80% diameter stenosis in the right coronary artery (RCA). A stent was respectively implanted in LCX and RCA (Fig. 1B). Postoperative ECG revealed that both inferior and V5–V6 leads ST segment were decreased (Fig. 1C).

After coronary intervention, we performed an echocardiography examination immediately. The results showed a significant decrease in ejection fraction (24%) and moderate mitral regurgitation due to mitral chordae tendineae rupture. Considering that the blood pressure of patient was still difficult to maintain after a large dose of norepinephrine treatment and at the same time the risk of cardiac arrest remains high, ECMO catheterization was carried out with venoarterial ECMO connection to maintain oxygenation. Subsequently, the indicators of patient were gradually improved. Three days later, the ECMO flow rate was reduced to 1.5 L/min and then the blood pressure, respiration, and heart rate of patient were stable. Meanwhile, the autonomic circulation test was acceptable. Therefore, ECMO was then deactivated.

**Fig. 2:**
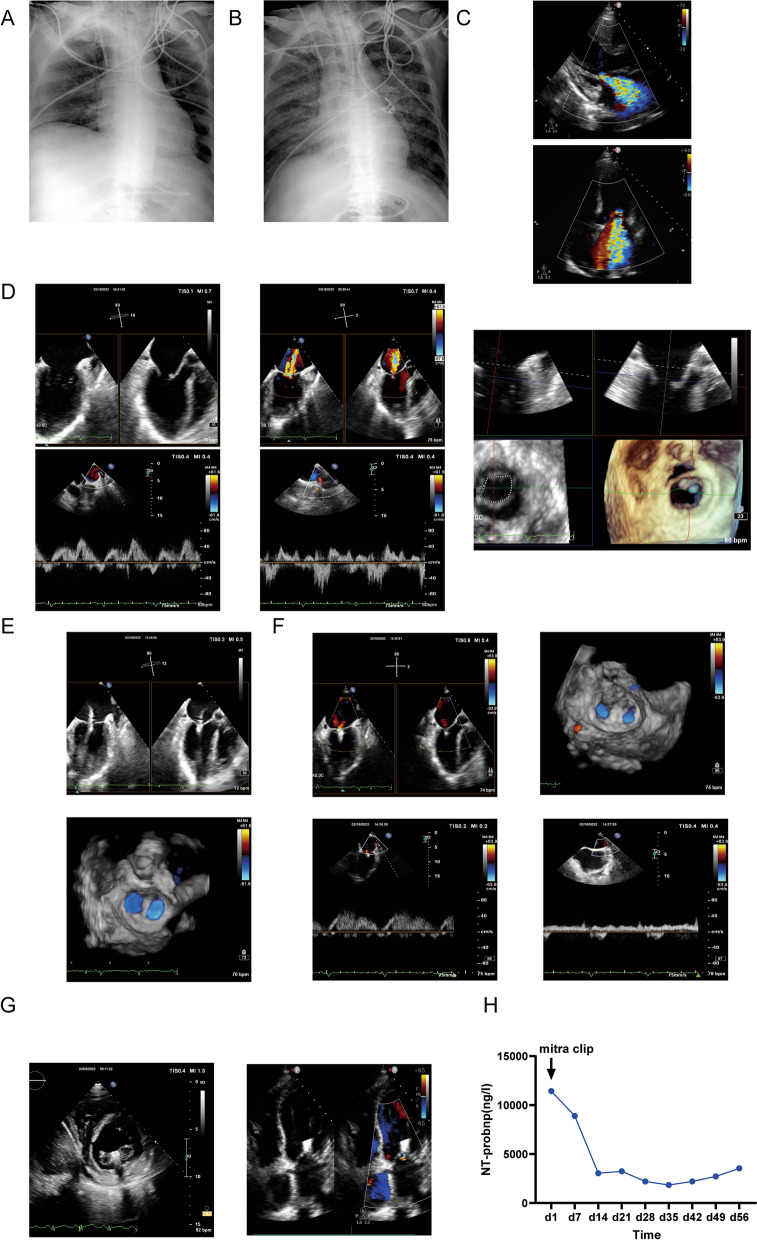
**A** Chest radiography at admission; **B** Chest radiography for review; **C** Cardiac ultrasound before operation; **D** Cardiac esophageal ultrasound and pulmonary venous flow pattern before operation; **E** Cardiac esophageal ultrasound after the first clip release. **F** Cardiac esophageal ultrasound and pulmonary venous flow pattern after the second clip release. **G** Cardiac ultrasound after operation; **H** NT-proBNP after operation

After stopping ECMO intervention, the patient developed chest tightness accompanied by high NT-proBNP and mitral systolic murmur. Compared with chest radiography at admission (Fig. 2A), the review chest radiography showed that pulmonary edema was significantly aggravated (Fig. 2B). The heart failure symptom in patient was not significantly improved after three months of standard medication (including diuretics, vasodilators, cardiotonic drugs and improved prognosis agents). Follow-up echocardiography indicated massive mitral regurgitation (Fig. 2C) and severe pulmonary hypertension (70 mmHg). Considering the severity of the patient's condition (STS risk score is 31.3%) and the wishes of family members, this patient was thus referred for emergency transcatheter mitral valve edge-to-edge repair with MitraClip. Esophageal echocardiography was used again to assess state of an illness before operation (Fig. 2D), which indicated a large amount of mitral regurgitation. The regurgitation area of mitral valve was 4.14 cm^2^, and the mean pressure gradient was 1 mmHg. Pulmonary venous flow pattern showed a reverse blood flow in pulmonary veins during systolic period. Under the guidance of esophageal ultrasound, the appropriate site was selected for puncture through atrial septum. The mitral valve clip was then delivered to the left atrium via the guidance system. As illustrated in Fig. 2E, the mitral valve was successfully clamped, but the results shows that there was still some reflux on the free edge side. Therefore, a second clip was placed based on the same procedures. Only a small amount of reflux was monitored via esophageal ultrasound (Fig. 2F). Pulmonary venous flow pattern showed that there was no reverse blood flow in pulmonary veins during systolic period. The symptom of chest tightness was significantly improved after operation and at the same time, mitral murmur was disappeared. Additionally, cardiac ultrasound re-examination indicated that mitral regurgitation was remarkably reduced (Fig. 2G), with the mitral valve regurgitation area of 1.96 cm^2^ and the mean pressure gradient of 2 mmHg. Moreover, the heart ejection fraction was back up to 50%, pulmonary artery pressure was normal, and NT-proBNP was also gradually decreased after operation (Fig. 2H).

## Discussion

As a leading cause of disability, the incidence of acute myocardial infarction is gradually increasing [4]. Although the mortality rate has decreased significantly with the development of reperfusion therapy [5], postoperative complications are still inevitable. Mitral regurgitation due to papillary muscle and chordae tendineae rupture may occur within two to seven days after acute myocardial infarction. Its symptoms generally manifest as sudden chest tightness, pulmonary edema, cardiogenic shock and other symptoms with recent systolic murmurs of the mitral valve. A recent study showed that the incidence of mitral regurgitation after acute myocardial infarction was 29%, in which mild regurgitation accounts for 76%, moderate regurgitation was for 21%, and severe regurgitation was for 3%. It has been proven that the presence of mitral regurgitation is closely associated with increasing mortality of acute myocardial infarction [6] and at the same time, the incidence of mitral regurgitation is higher for ST-segment elevation myocardial infarction [7]. Current studies suggest that the mechanism of ischemic mitral regurgitation is mainly because of the imbalance between the increased tethering force and the decreased closing force. The left ventricular contractile force was decreased, the synchronization between the two papillary muscles was decreased, and the left ventricular was not synchronized as a whole, resulting in the reduction of the closing force. The increase in tethering force was mainly because of the displacement of the posterior papillary muscle apical, posterior and lateral, and the distortion of the left ventricle after infarction [8]. Early intervention and surgical treatment are essential for these patients with mitral regurgitation. However, surgical mitral valve repair or replacement often undergoes high-risk. In the SHOCK clinical trial, surgical treatment of mitral regurgitation after myocardial infarction has a mortality rate of up to 39% [9].

MitraClip, originated from the edge-to-edge restoration technique proposed by Italian doctor Ottavio Alfieri [10], is one of important methods for the interventional treatment of mitral regurgitation and has been used in more than 100,000 cases worldwide. Not only was the first successful animal experiment reported in 2003 [11], but MitraClip was also successful in human trials [12]. Although the MITRA-FR Clinical Trials showed that there were no significant differences in the 1-year rate of death or unplanned hospitalization due to heart failure between patients who underwent percutaneous mitral valve repair while receiving drug therapy and those who received drug therapy alone in patients with severe secondary mitral regurgitation [13], MitraClip intervention significantly reduced rehospitalization and mortality rates in patients with heart failure symptoms and severe mitral regurgitation, according to COAPT registry studies [14]. By comparing these two previous studies, it was found that patients with mitral regurgitation were more severe in the COAPT study than those in the MITRA-FR study, thus providing a possible explanation for the different results of the two clinical trials [15]. Additionally, Benito-Gonzalez et al. evaluated the efficacy of MitraClip in patients with acute-decompensated heart failure (ADHF) and found that MitraClip implantation could be used as an emergency treatment during admission [16]. Musuku et al. also found that emergency MitraClip was theoretically feasible for patients with severe mitral regurgitation [17]. However, the risk of short- and intermediate-term adverse outcomes after emergency MitraClip remained relatively high and may lead to increased mortality for cardiovascular diseases [17]. The research data of MitraClip interventional therapy in the Asia–Pacific region were released in 2016, demonstrating that MitraClip is a safe and effective choice to significantly improve both degenerative and functional mitral regurgitation [18]. Although the effects of MitraClip implantation on chronic severe symptom secondary mitral regurgitation have been validated, there is still little data on patients with severe acute ischemic secondary mitral regurgitation. Haberman et al. concluded that MitraClip significantly reduces mitral regurgitation and improves hemodynamic parameters in 20 patients with acute myocardial infarction [19]. Meanwhile, in another study conducted by Haberman et al. in 2022, it was shown that the in-hospital mortality and 1-year mortality of patients with mitral regurgitation undergoing surgical treatment were significantly higher relative to those undergoing MitraClip, suggesting that MitraClip can replace surgery in the treatment of mitral regurgitation after myocardial infarction [20].

In our case report, this patient with cardiac arrest caused by acute myocardial infarction was successfully rescued by emergency coronary intervention and ECMO support. However, drug treatments had not significantly improve the subsequent mechanical complications of mitral regurgitation. MitraClip intervention was immediately carried out after discussion with the patient’s family members. After the MitraClip treatment, heart failure symptoms were significantly improved, NT-probNP was dramatically decreased, and mitral systolic murmurs were disappeared. Additionally, an echocardiography re-evaluation revealed a significant decrease in mitral regurgitation.

## Conclusion

This case suggests that MitraClip is a safe and effective treatment for post-myocardial infarction mitral regurgitation. At present, MitraClip was successfully launched in China in 2020 and is still being developed. We believe that MitraClip will be further applied in patients with mitral regurgitation who cannot tolerate surgery in the near future.

## Data Availability

We declared that materials described in the manuscript, including all relevantraw data, will be freely available to any scientist wishing to use them for noncommercial purposes.

## References

[1] López-Pérez M, Estévez-Loureiro R, López-Sainz A, Couto-Mallón D, Soler-Martin MR, Bouzas-Mosquera A (2014). Long-term prognostic value of mitral regurgitation in patients with ST-segment elevation myocardial infarction treated by primary percutaneous coronary intervention. Am J Cardiol.

[2] Vahanian A, Beyersdorf F, Praz F, Milojevic M, Baldus S, Bauersachs J (2021). ESC/EACTS Guidelines for the management of valvular heart disease. Eur Heart J.

[3] O'Gara P, Calhoon J, Moon M, Tommaso C (2014). American College of Cardiology, American Association for Thoracic Surgery, et al.Transcatheter therapies for mitral regurgitation: a professional society overview from the american college of cardiology, the american association for thoracic surgery, society for cardiovascular angiography and interventions foundation, and the society of thoracic surgeons. Catheter Cardiovasc Interv.

[4] Bhatt D, Lopes R, Harrington RJJ (2022). Diagnosis and treatment of acute coronary syndromes: a review. JAMA.

[5] Thiele H, Ohman E, de Waha-Thiele S, Zeymer U, Desch S (2019). Management of cardiogenic shock complicating myocardial infarction: an update 2019. Eur Heart J.

[6] Sharma H, Radhakrishnan A, Nightingale P, Brown S, May J, O'Connor K (2021). Mitral regurgitation following acute myocardial infarction treated by percutaneous coronary intervention-prevalence, risk factors, and predictors of outcome. Am J Cardiol.

[7] Nishino S, Watanabe N, Kimura T, Enriquez-Sarano M, Nakama T, Furugen M (2016). The course of ischemic mitral regurgitation in acute myocardial infarction after primary percutaneous coronary intervention: from emergency room to long-term follow-up. Circ Cardiovasc Imaging.

[8] Piérard LA, Carabello BA (2010). Ischaemic mitral regurgitation: pathophysiology, outcomes and the conundrum of treatment. Eur Heart J.

[9] Hochman JS, Buller CE, Sleeper LA, Boland J, Dzavik V, Sanborn TA (2000). Cardiogenic shock complicating acute myocardial infarction–etiologies, management and outcome: a report from the SHOCK Trial Registry: SHould we emergently revascularize Occluded Coronaries for cardiogenic shocK?. J Am Coll Cardiol.

[10] Maisano F, Caldarola A, Blasio A, De Bonis M, La Canna G, Alfieri O (2003). Midterm results of edge-to-edge mitral valve repair without annuloplasty. J Thorac Cardiovasc Surg.

[11] St Goar F, Fann J, Komtebedde J, Foster E, Oz M, Fogarty T (2003). Endovascular edge-to-edge mitral valve repair: short-term results in a porcine model. Circulation.

[12] Condado J, Acquatella H, Rodriguez L, Whitlow P, Vélez-Gimo M, St Goar FG (2006). Percutaneous edge-to-edge mitral valve repair: 2-year follow-up in the first human case. Catheter Cardiovasc Interv.

[13] Obadia JF, Messika-Zeitoun D, Leurent G, Iung B, Bonnet G, Piriou N (2018). Percutaneous repair or medical treatment for secondary mitral regurgitation. N Engl J Med.

[14] Stone GW, Lindenfeld J, Abraham WT, Kar S, Lim DS, Mishell JM (2018). Transcatheter mitral-valve repair in patients with heart failure. N Engl J Med.

[15] Gaasch William H, Aurigemma Gerard P, Meyer Theo E (2020). An appraisal of the association of clinical outcomes with the severity of regurgitant volume relative to end-diastolic volume in patients with secondary mitral regurgitation. JAMA Cardiol.

[16] Benito-González T, Estévez-Loureiro R, Del Castillo S, Minguito-Carazo C, Garrote-Coloma C, Alonso-Rodríguez D (2021). Clinical outcomes following urgent vs. elective percutaneous mitral valve repair. Cardiovasc Revasc Med.

[17] Musuku SR, Mustafa M, Pulavarthi M, Doshi I, Zhang Y, Luu S (2022). Procedural, short-term, and intermediate-term outcomes in propensity-matched patients with severe mitral valve regurgitation undergoing urgent versus elective mitraclip percutaneous mitral valve repair. J Cardiothorac Vasc Anesth.

[18] Tay E, Muda N, Yap J, Muller D, Santoso T, Walters D (2016). The MitraClip Asia-pacific registry: differences in outcomes between functional and degenerative mitral regurgitation. Catheter Cardiovasc Interv.

[19] Haberman D, Taramasso M, Czarnecki A, Kerner A, Chrissoheris M, Spargias K (2019). Salvage MitraClip in severe secondary mitral regurgitation complicating acute myocardial infarction: data from a multicentre international study. Eur J Heart Fail.

[20] Haberman D, Estévez-Loureiro R, Benito-Gonzalez T, Denti P, Arzamendi D, Adamo M (2022). Conservative, surgical, and percutaneous treatment for mitral regurgitation shortly after acute myocardial infarction. Eur Heart J.

